# Involvement of the central amygdaloid nucleus in the regulation of sex differences in the stress relief response in mice

**DOI:** 10.1186/s13293-025-00819-z

**Published:** 2026-01-16

**Authors:** Yujia Li, Jialing Xie, Jie Chen, Xiaofei Huo, Meng Wang, Tao Hu, Manfei Deng, Wenyu Cao, Yang Xu

**Affiliations:** 1 Institute of Neuroscience, Hengyang Medical School, University of South China, Hengyang, 421001 Hunan China; 2 Clinical Anatomy & Reproductive Medicine Application Institute, Hengyang Medical School, University of South China, Hengyang, 421001 Hunan China

**Keywords:** Stress relief, Natural reward, Conditioned place preference, Sex differences, Central amygdala (CeA)

## Abstract

**Background:**

Sex differences in brain function critically influence vulnerability to stress-related disorders such as anxiety and depression. Stress relief, defined as a positive emotional state following the termination of a threat, has been proposed as a natural reward promoting resilience. However, little is known about sex differences in stress relief behavior and the underlying neural mechanisms involved.

**Methods:**

Adult male and female C57BL/6J mice were subjected to a conditioned place preference (CPP) paradigm to evaluate stress relief responses following acute restraint stress. Estrous cycle stages in females were monitored during the test day to exclude hormonal effects. Whole-brain neuronal activity was assessed using large-scale c-Fos mapping to identify sex-specific neural correlates of stress relief. To establish causality, chemogenetic manipulations were performed by bilaterally expressing hM3Dq or hM4Di DREADDs in the central amygdala (CeA), followed by clozapine-N-oxide administration to selectively activate or inhibit CeA neurons during the behavioral test.

**Results:**

We found that male mice exhibited a robust stress relief response, whereas female mice failed to display stress relief, independent of stress intensity or the estrous cycle. c-Fos mapping revealed CeA neuronal inactivation in males but not females during stress relief. Chemogenetic activation of CeA neurons abolished stress relief in males, whereas inhibition of CeA neurons facilitated stress relief in females.

**Conclusions:**

These data highlight the sex-specific role of CeA neurons in regulating stress relief, with inactivation promoting relief in males and inhibition enabling relief in females. These findings may provide a neural basis for understanding sex-specific mechanisms of stress relief and offer insights into the circuit-level origins of sex-biased vulnerability to stress-related psychiatric disorders.

**Highlights:**

- Male mice exhibited robust stress relief responses, whereas females failed to display stress relief.
- The absence of stress relief in females was independent of stress intensity and estrous cycle stage.
- Whole-brain c-Fos mapping revealed sex-specific neural activation patterns, with CeA neurons inactivated in males but not in females during stress relief.
- Chemogenetic activation of CeA neurons abolished stress relief in males, whereas inhibition of CeA neurons enabled stress relief in females.

**Plain English Summary:**

Exploring sex differences in the brain is important for understanding the effects of such differences in stress-related disorders characterized by sex bias, as well as their therapeutic implications. In this manuscript, we examined sex differences in stress relief, a positive emotion triggered by the absence of an expected threat or the termination of an ongoing threat, as well as its potential mechanism. Our findings revealed sex variation in the stress relief response, as female mice did not show a stress relief response, which was independent of stress intensity or estrous cycle fluctuations. By determining neuronal activation in the mouse brain following a stress relief assay, we found that CeA neurons are inactivated during this paradigm in male mice but not in female mice. Moreover, chemogenetic activation of CeA neurons abolished the stress relief responses in male mice, whereas chemogenetic inactivation of CeA neurons facilitated the stress relief responses in female mice. We speculated that CeA neurons might play a key role in the sexually dimorphic stress relief response in mice. Hence, it is important to consider sex differences in both preclinical and clinical research studies that attempt to understand the mechanism related to stress relief.

**Supplementary Information:**

The online version contains supplementary material available at 10.1186/s13293-025-00819-z.

## Introduction

Sex differences in both brain function and pathology are important areas of research because they may influence not only normal brain function but also disease outcomes [1, 2]. In fact, accumulating evidence shows that sexual dimorphism at the level of the central nervous system might affect many biological activities and behaviors, including stress-related responses, cognitive performance and pain [3–5]. Notably, women are more susceptible to stress-related disorders such as anxiety and depression [6–8], characterized by dysfunction of corticolimbic brain regions (e.g., the medial prefrontal cortex, amygdala and hippocampus) that are critical for emotion regulation and cognitive function [9–11]. Therefore, understanding the mechanisms underlying sex differences in stress responses might help us better understand the basis of the sex-biased prevalence and presentation of neuropsychiatric conditions.

Stress relief, an evolutionarily conserved phenomenon, is a positive emotion triggered by the absence of an expected threat or the termination of an ongoing threat [12–14]. Notably, the relief of aversive states, including stress or pain, often promotes a positive emotional state [15]; therefore, stress relief is considered to be a natural reward to both humans and animals [16]. In contrast, people with anhedonic symptoms report blunted stress relief at threat omission and exhibit reduced active avoidance [17]. A recent study revealed that stress relief could counteract the detrimental effects of stress determined by the conditioned place preference (CPP) test [18]. Furthermore, the dopamine (DA) system is suggested to be activated and required for the stress relief response in this behavioral paradigm [18, 19]. Although these findings provide important insights into the mechanism underlying stress relief and depression, we still know relatively little about the potential sex differences in this behavior. Despite their biological importance, the brain circuits regulating stress relief remain poorly understood, particularly with respect to sex differences.

To gain a comprehensive understanding of stress relief, it is imperative to systematically compare the intrinsic properties between not only different brain subregions but also different sexes. The present research extended previous findings [18]; we observed sex variation in the stress relief response, as female mice did not show a stress relief response, which was independent of stress intensity or estrous cycle fluctuations. By applying a large-scale c-Fos brain mapping approach following a stress relief assay, we found that neurons in the amygdala, especially the CeA, are inactivated during this paradigm in male mice but not in female mice. Moreover, chemogenetic activation of CeA neurons abolished the stress relief responses in male mice, whereas chemogenetic inactivation of CeA neurons facilitated the stress relief responses in female mice. Considering previous findings reporting that the amygdala is involved in the regulation of behavior flexibility [20, 21], we speculated that CeA neurons might play a key role in the sexually dimorphic stress relief response in mice. Our data might contribute to the increased understanding of sex differences in stress relief, providing essential insight into how specific brain regions contribute to sex disparities in stress-related mental illnesses.

## Materials and methods

### Animals

Eight-week-old male and female C57BL/6J mice were obtained from the Hunan SJA Lab Animal Center of Changsha (Hunan, China). All the animals were housed at a controlled temperature (22 ± 2 °C) under a 12-h light/dark cycle with free access to food and water. The protocol was approved by the Animal Care and Use Committee of the University of South China and was in compliance with the National Institutes of Health Guide on Laboratory Animals.

### Stress relief behavioral test: conditioned place preference (CPP)

The test apparatus was a light/dark box with a door between two chambers (20 cm×20 cm×20 cm) (Shanghai Xinruan Information Technology Co., Ltd.). The illumination status in each compartment was regulated by the overhead lighting switches (light box: 200–400 lx; dark box: less than or equal to 5 lx). The CPP test was carried out according to a previous study [18]. Briefly, mice were subjected to stress and immediately placed in the light chamber of the CPP apparatus, with the door closed, for 30 min on the first day. On the second day, this procedure was repeated following stress exposure to allow the mice to associate the light chamber with post-stress relief. On the third day, the mice were allowed to freely explore both the light and dark chambers to assess their chamber preference [18]. Mice that had previously experienced stress were expected to spend more time in the light chamber, indicating that the light chamber was associated with a rewarding experience and the subsequent relief from stress. In contrast, control mice that were not exposed to stress were anticipated to show a preference for the dark chamber.

### Restraint stress relief

Mice were restrained in a 50-mL tube (with holes for air flow, 2.5 cm inside diameter, 10 cm length) for 2 h. Immediately after stress, the mice were placed into the light box with the door shut for 30 min. This procedure was repeated on two successive days. On Day 3, the animals were allowed to freely explore the light/dark box with the door open for 15 min. Control mice underwent the same procedure and were placed in the light chamber for the same duration on Days 1 and 2 but without prior restraint stress and were similarly tested on Day 3. After each training or testing session, the apparatus was cleaned with 75% ethanol. Video cameras positioned directly above the chambers were used to track each mouse via SuperMaze software (SuperMaze+, Shanghai Xinruan Information Technology Co., Ltd.). The CPP score was calculated as follows: Time spent in the light chamber – Time spent in the dark chamber.

### Foot shock stress relief

Mice were categorized into three groups and placed in a fear conditioning apparatus (30 cm×30 cm×50 cm), where they received foot shock stimuli (10 shocks, 0.5 mA, 2 s duration) delivered at regular intervals over a period of 5 min. Immediately after stress, the mice were placed into the light box with the door shut for 30 min, and this training lasted for 2 days. On Day 3, the animals were allowed to freely explore the light/dark box with the door open for 15 min. Control mice experienced all the training and testing but without foot shock stress. After each training or testing session, the apparatus was cleaned with 75% ethanol. Video cameras positioned directly above the chambers were used to track each mouse via SuperMaze software (SuperMaze+, Shanghai Xinruan Information Technology Co., Ltd.). The CPP score was calculated as follows: Time spent in the light chamber – Time spent in the dark chamber.

To explore whether the strength of foot shock stress affects female mice’s CPP, the animals received different regular foot shock stimuli (10 times, 0.5 mA, 2 s duration; 5 times, 0.5 mA, 2 s duration; 10 times, 0.2 mA, 2 s duration) over a period of 5 min. Immediately after stress, the mice were placed into the light box with the door closed for 30 min, and this training lasted for 2 days. On Day 3, the animals were allowed to freely explore the light/dark box with the door open for 15 min. Control mice underwent the same procedure and were placed in the light chamber for the same duration on Day 1 and 2 but without prior foot shock stress and were similarly tested on Day 3. After each training or testing session, the apparatus was cleaned with 75% ethanol. Video cameras positioned directly above the chambers were used to track each mouse via SuperMaze software (SuperMaze+, Shanghai Xinruan Information Technology Co., Ltd.). The CPP score was calculated as follows: Time spent in the light chamber –Time spent in the dark chamber.

### Immunohistochemistry

One and a half hours after the CPP test, 3 mice from each group were sacrificed for immunohistochemistry, which was carried out as described in our previous study [22]. After being anesthetized with 10% sodium pentobarbital (100 mg/kg), the animals were transcardially perfused with normal saline, followed by 4% (w/v) paraformaldehyde. Then, the brains were removed and postfixed in 4% paraformaldehyde (PFA) in phosphate-buffered saline (PBS) at 4 °C overnight, after which they were dehydrated in 15% and 30% sucrose solutions at 4 °C. A cryostat was used to cut the brains into 30 μm thick sections. To remove endogenous peroxidase, the sections were treated with 3% H_2_O_2_ for 25 min, and the free-floating sections were then washed with 0.01 M phosphate-buffered saline (PBS) three times (10 min each time). The sections were then blocked with 5% goat serum (with 0.1% Triton X-100) for 2 h at room temperature and incubated with a rabbit anti-c-Fos antibody (1:1000, CST) for 2 h at room temperature before they were incubated at 4 °C overnight. On the second day, the sections were washed with 0.01 M PBS three times (10 min each time) and then incubated with secondary reagents containing biotinylated goat anti-rabbit immunoglobulin (Proteintech) for 2 h at room temperature. After being washed three times (10 min each), the sections were further incubated with an avidin-biotin peroxidase complex (Vector ABC Kit) for 2 h at room temperature. After washing, the peroxidase reaction proceeded using a diaminobenzidine substrate (ZSGB-BIO) prepared according to the manufacturer’s instructions. All sections were mounted onto gelatin-coated slides, dehydrated in a graded ethanol series and xylene and then cover slipped. All the images were captured using an optical microscope (EX30, Shunyu, China) under identical conditions. Cells immunopositive for c-Fos were counted by a researcher who was blinded to the treatment.

The colocalization of GFP with the neuronal activation marker c-Fos was carried out as described in our previous study [23]. Briefly, after being blocked with 5% goat serum (with 0.2% Triton) on a shaker at room temperature for 2 h, the slices were incubated with a primary antibody against c-Fos (rabbit c-Fos, 1:1000, CST) on a shaker at room temperature for 2 h, followed by incubation at 4 ℃ overnight. After being rewarmed on a shaker at room temperature for 30 min the next day, the slices were washed with 0.01 M PBS 3 times for 10 min each. The slices were then incubated with Alexa 594-labeled goat anti-rabbit (1:1000, Proteintech) on a shaker at room temperature for 1 h. After being washed 3 times with 0.01 M PBS (10 min each), the slices were mounted and covered with immunofluorescence buffer containing DAPI (Solarbio). All the images were captured under the same conditions using a ZEISS fluorescence microscope equipped with a 20× objective (ZEISS, Germany). Brain sections were collected at the following rostrocaudal levels according to the Mouse Brain Atlas: approximately Bregma − 1.70 mm to − 1.94 mm.

### Estrous cycle staging cytology

Vaginal smears were collected using a pipette containing normal saline (NS) and gently leaned toward or inserted into the vagina of restrained female mice. Mucous tissue was then trickled onto dry glass slides and stained with hematoxylin (Servicebio G1005-1) for 2 min, followed by staining with eosin (Servicebio G1005-2) for 30 s [24]. All the images were observed and captured using an optical microscope (EX30, Shunyu, China) under identical conditions.

Vaginal cytological assessments categorized the estrous cycle into distinct phases as follows: proestrus, characterized by a predominance of rounded nucleated epithelial cells; estrus, identified by a majority of cornified cells; early diestrus, marked by a preponderance of leukocytes with distinctly lobulated nuclei; and late diestrus, defined by a reduced number of cells compared with early diestrus, with nuclei appearing in clusters, and the presence of amorphous, degenerating leukocytes [25]. Periods characterized by lower sex hormone levels were designated the D1/D2 phases, whereas periods with elevated sex hormone levels were referred to as the P/E phases [26].

### Stereotaxic surgery

The mice were anesthetized using 10% sodium pentobarbital (45 mg/kg, i.p.) solution and placed in a prone position in a stereotaxtic apparatus for virus injection, followed by our previously published methodology [27]. The mice were subjected to a pinch test to ensure the depth of anesthesia. After the mice lost consciousness, they were fixed on the stereotaxtic frame with a nose clamp and ear bars. After the fur was shaved, a longitudinal incision was made to expose the bregma. Two burr holes were drilled on the two sides of the skull at 1.2 mm lateral to the midline and 2.9 mm posterior to the bregma using a high-speed stereotaxic drill.

AAV2/9-hSyn-hM3D(Gq)-ER2-P2A-EGFP-WPRE-pA (Titer: 1.29 × 10^13^ v.g./ml) or AAV2/9-hSyn-hM4D(Gi)-ER2-P2A-EGFP-WPRE-pA (Titer: 2.01 × 10^13^ v.g./ml) was purchased from Taitool Bioscience Co. Ltd. (Shanghai, China). For chemogenetic manipulation, 200 nL of virus was bilaterally injected into the central amygdala (CeA) of mice using a microsyringe (RWD, 79014) at a rate of 100 nL/min (coordinates: AP − 1.2 mm, ML ± 2.90 mm, and DV − 5.30 mm). Ten min after the injection, the microsyringe was slowly withdrawn, and the incision was sutured. The mice were allowed to recover in a warmed cage with food and water before being returned to their home cages. Three weeks after viral injection, the mice received an intraperitoneal injection of clozapine-N-oxide (CNO, 1 mg/kg) (Tocris), which was diluted in saline to the desired concentration, 30 min prior to the CPP test on Day 3 [28]. Control mice were injected with the same virus but received an equivalent volume of saline 30 min before the CPP test to account for the potential effects of viral expression alone.

### Statistical analyses

All the statistics were analyzed using GraphPad Prism 5.0 (GraphPad Software, San Diego, CA, USA). The data are presented as the mean ± SEM. The initial sample sizes in the behavioral and biochemistry studies were based on previous studies using similar experimental designs [18]. The results show the precise size of the sample, the statistical analysis used, and the test results for each experiment. Only the animals with the correct injection or enhanced c-Fos expression were included in the analysis of the chemogenetic manipulation experiment results. For c-Fos quantification, c-Fos-positive cells were counted bilaterally in selected brain regions (three sections per mouse, three mice per group). Each hemisphere was considered one sample. Sections without clearly defined target regions were excluded from quantification. For experiments involving multiple factors, such as SEX and STRESS, a two-way ANOVA was performed to assess the main effects and interactions. When significant interactions were detected, post hoc multiple comparisons were performed using the Bonferroni correction to control for type I errors. For two-group comparisons, Student’s t test was used. When unequal variances were detected by the F-test, Welch’s correction was applied. Statistical significance was set at *p* < 0.05.

## Results

### Stress relief shows a sex difference in restraint-stressed mice

**Fig. 1:**
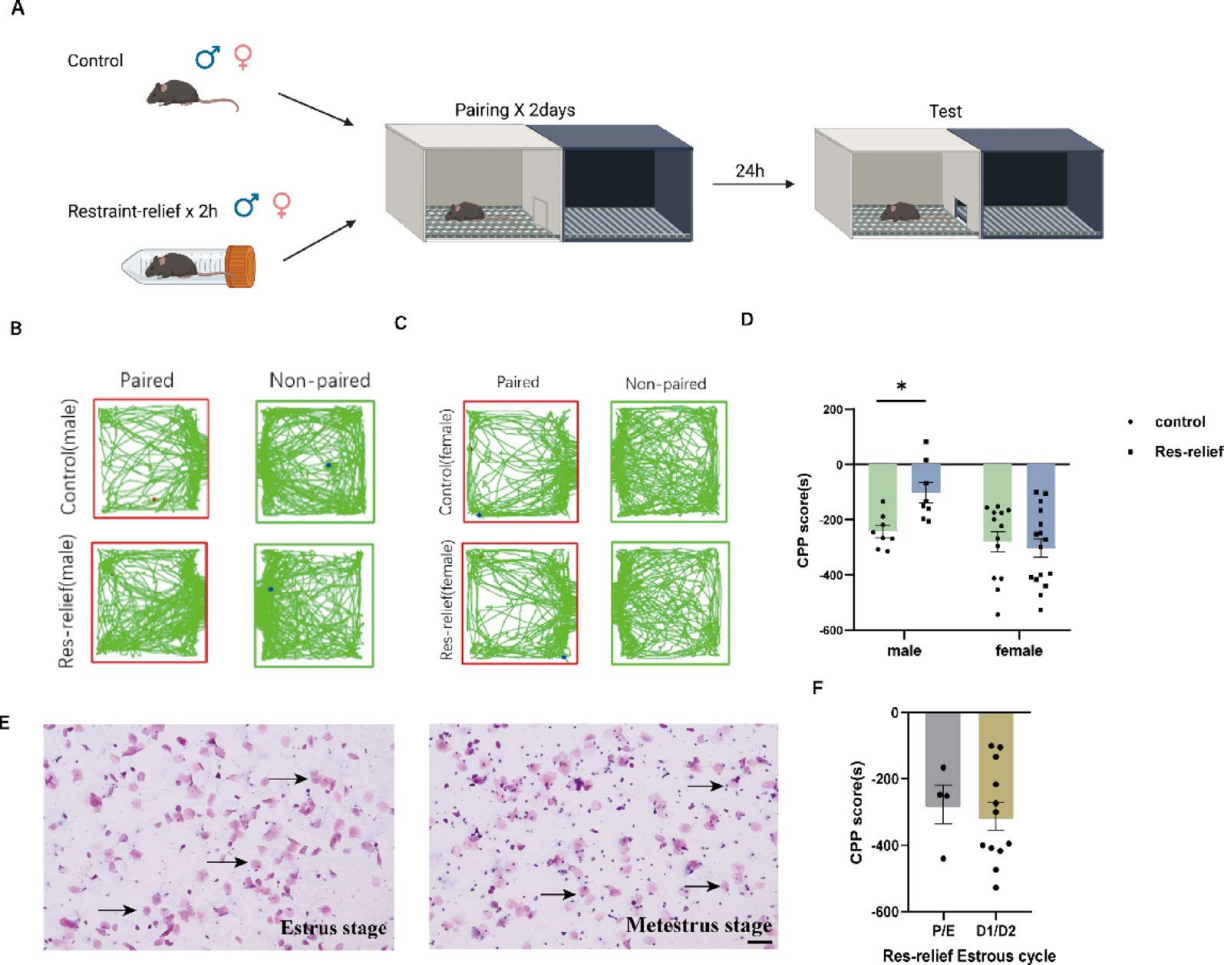
Sex difference in stress relief among restraint-stressed mice. **(A)** Schematic timeline of the restraint-relief CPP test. **(B)** Trajectory map of male mice in the restraint-relief CPP test. **(C)** Trajectory map of female mice in the restraint-relief CPP test. **(D)** CPP scores of male mice and female mice in the restraint-relief CPP test (Control male = 8, Res-relief male = 8; Control female = 13, Res-relief female = 16). **(E)** Representative images of the estrous cycle in female restraint-relief mice (bar = 100 μm). **(F)** Phase statistics of the estrous cycle from female mice (P/E = 4, D1/D2 = 12). Data are presented as the mean ± SEM and were analyzed by unpaired two-tailed Student’s t test (for two-group comparisons) or two-way ANOVA followed by Bonferroni’s post hoc correction. ^*^p < 0.05 versus control males

Although stress relief has been shown to evoke strong and rewarding effects in male mice [18], whether this is the same in females is unknown. Therefore, both male and female mice were subjected to the restraint stress relief protocol (Fig. 1A). Compared with control male mice, male mice that experienced restraint stress exhibited a stronger preference for the light box (Fig. 1B). Intriguingly, female mice did not display such a preference (Fig. 1C). Two-way ANOVA revealed a significant main effect of sex (F_(1,41)_ = 9.985, *p* = 0.0030) but not of restraint stress (F_(1,41)_ = 2.435, *p* = 0.1263). Notably, a significant sex × restraint stress interaction was detected (F_(1,41)_ = 4.827, *p* = 0.0337). Bonferroni’s multiple comparisons test revealed that stressed male mice had increased CPP scores (*p* = 0.0473), whereas no significant effect was observed in stressed female mice (*p* > 0.05) (Fig. 1D). To exclude the potential influence of the estrous cycle on the behavioral responses of female mice, we assessed the estrous cycle stage of female mice on the day when the stress relief test was conducted (Fig. 1E). However, no such effect was observed in the present study, as indicated by the same CPP scores among different estrous cycles (t _(14)_ = 0.4432, *p* = 0.6644) (Fig. 1F).

### Stress relief shows a sex difference in foot shock-stressed mice

**Fig. 2:**
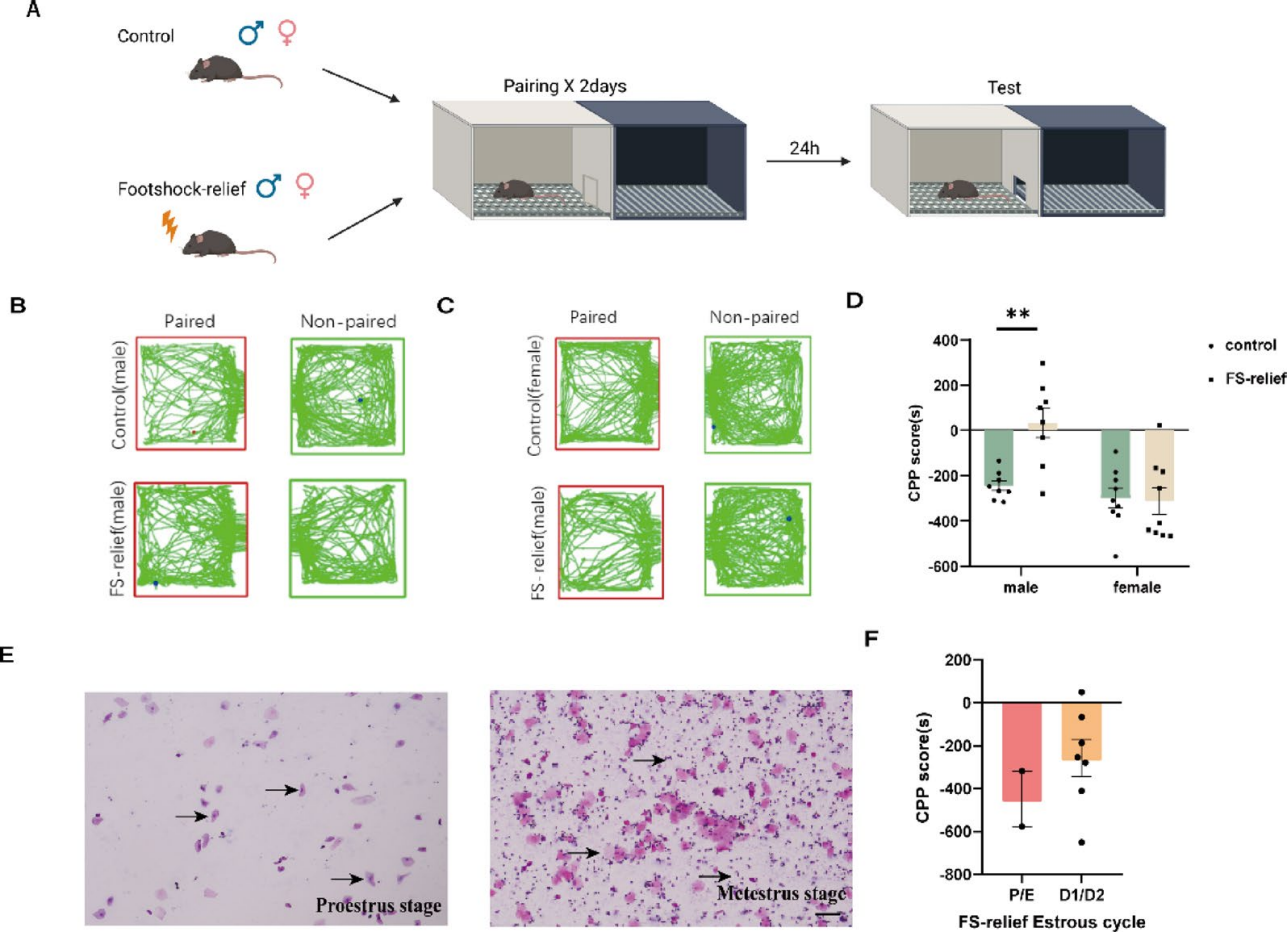
Sex difference in stress relief among foot shock mice. **(A)** Schematic timeline of the foot shock-relief CPP test. **(B)** Trajectory map of male mice in the foot shock-relief CPP test. **(C)** Trajectory map of female mice in the foot shock-relief CPP test. **(D)** CPP scores of male mice and female mice in the foot shock-relief CPP test (Control male = 8; Foot shock-relief male = 8; Control female = 9; Foot shock-relief female = 9). **(E)** Representative images of the estrous cycle of female mice with foot shock-relief (bar = 100 μm). **(F)** Phase statistics of the estrous cycle in female mice with foot shock-relief (P/E = 2, D1/D2 = 7). Data are presented as the mean ± SEM and were analyzed by unpaired two-tailed Student’s t test (for two-group comparisons) or two-way ANOVA followed by Bonferroni’s post hoc correction. ^**^p < 0.01 versus control males

We also used another cohort of male and female mice to test whether the same data were obtained for foot shock-induced stress relief behavior [18] (Fig. 2A). As expected, the male mice indeed showed a strong preference for the light box (Fig. 2B). However, female mice showed no preference for the light box (Fig. 2C). Two-way ANOVA revealed significant main effects of sex (F _(1,30)_ = 15.64, *p* = 0.0004) and foot shock (F_(1,30)_ = 6.761, *p* = 0.0143), as well as a significant sex × stress relief interaction (F_(1,30)_ = 8.250, *p* = 0.0074). Subsequent Bonferroni’s multiple comparisons test revealed that stressed male mice had increased CPP scores (*p* = 0.0015), whereas stressed female mice had the same CPP scores as control mice did (*p* > 0.05) (Fig. 2D). Similarly, no significant differences in CPP scores were observed across estrous cycle phases, indicating that the stress relief response in female mice is not phase-specific. (t_(7)_ = 0.8543, *p* = 0.4212) (Fig. 2E–F).

**Fig. 3:**
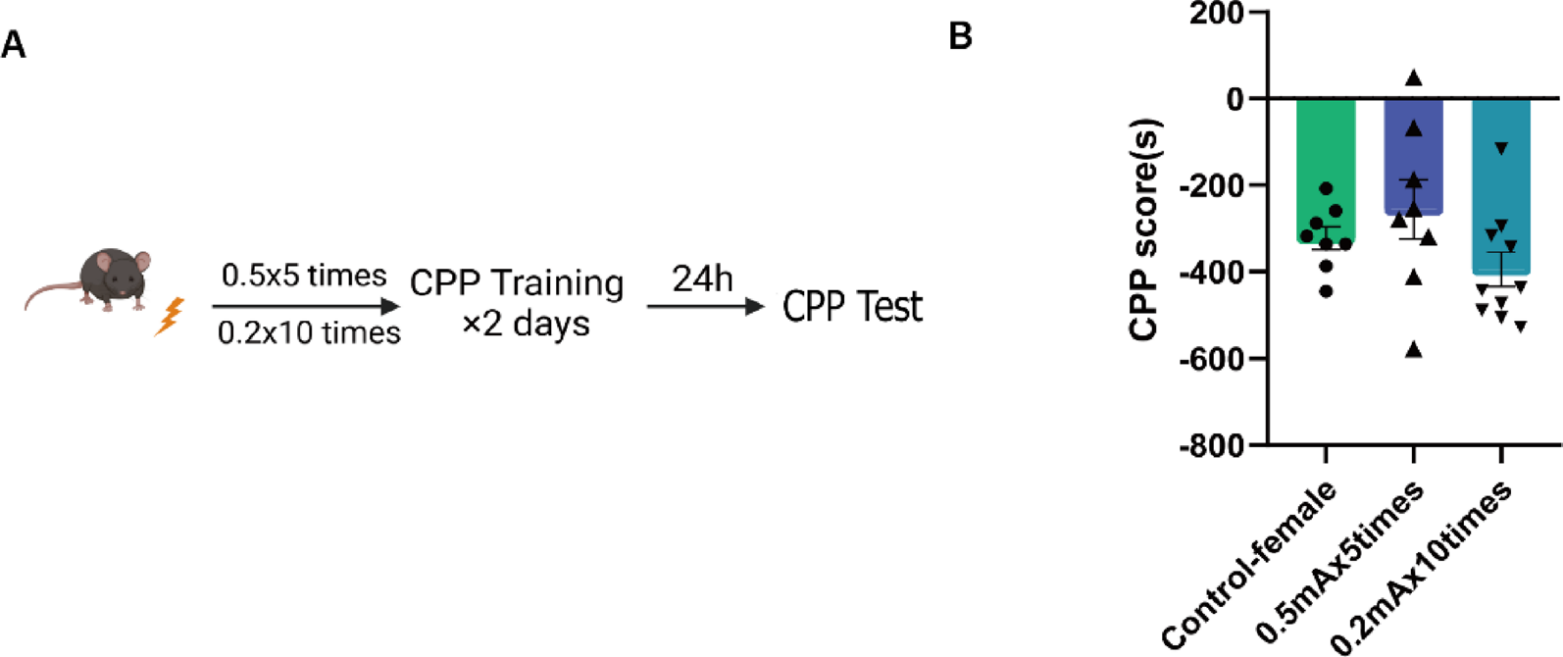
Female mice show no preference in a mild foot shock-relief CPP test. **(A)** Schematic timeline of the mild foot shock-relief CPP test. **(B)** CPP score of female mouse foot shock relief (control-female = 8; 0.5 mA×5 times = 8; 0.2 mA×10 times = 10. The data are presented as the mean ± SEM and were analyzed by one-way ANOVA followed by Bonferroni post hoc correction

Given that the stress sensitivity of male and female mice differed, we subsequently used another cohort of female mice to determine whether mild foot shock led to a preference for the light box among the female mice (Fig. 3A). However, one-way ANOVA revealed no significant difference among the three groups (F (2, 23) = 2.217, *p* = 0.1317) (Fig. 3B).

### Different neuronal activation patterns in the brains of male and female mice after stress relief

**Fig. 4:**
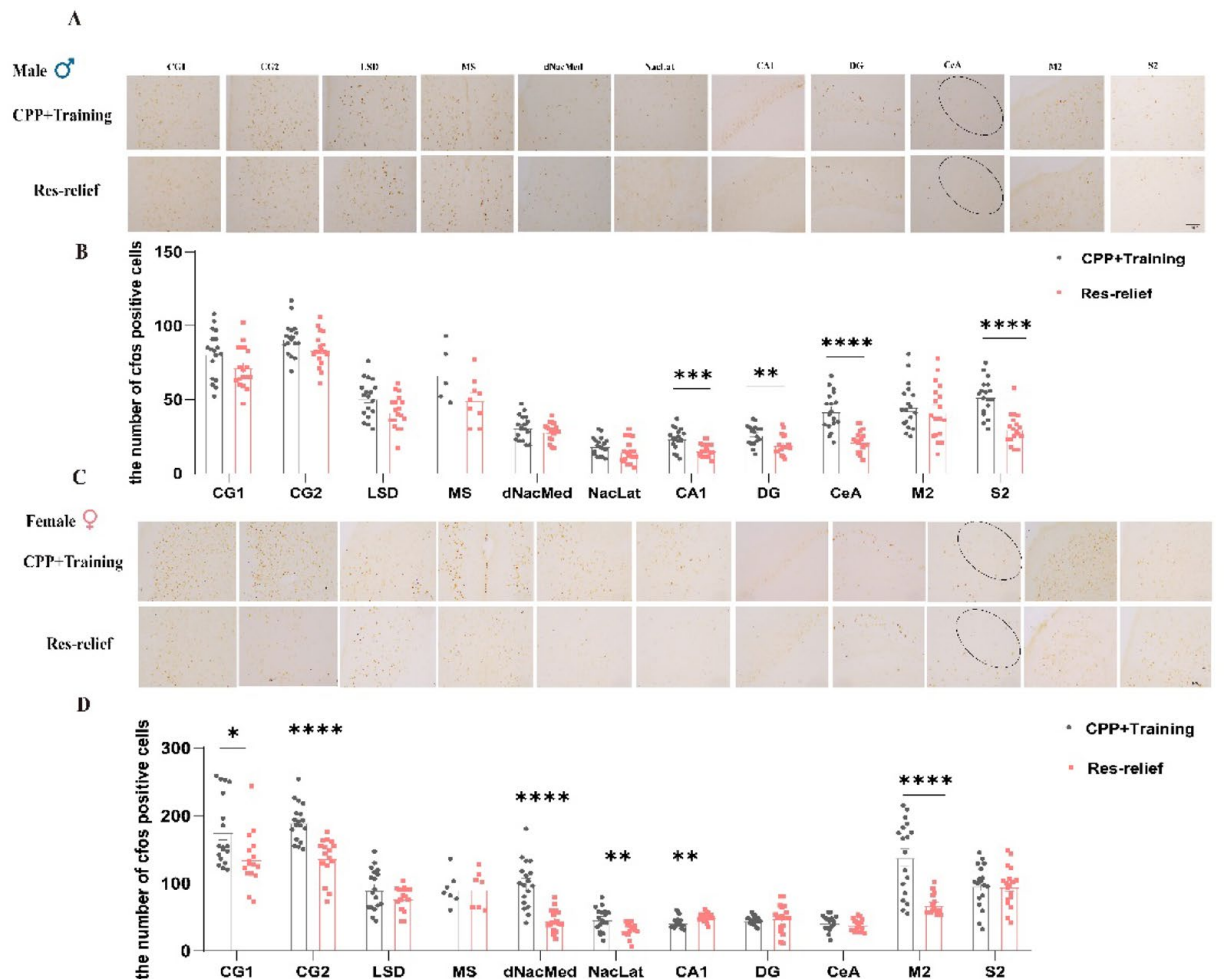
Different neuronal activation patterns in the brains of male and female mice after stress relief. **(A)** Representative c-Fos immunohistochemistry images of the PFC, LSD, MS, dNacMed, NacLat, CA1, DG, CeA, M2, and S2 in male mice after stress relief (bar = 100 μm) (*n* = 3). **(B)** Numbers of c-Fos-positive cells in the above brain regions. c-Fos-positive cells were quantified bilaterally (three sections per mouse, three mice per group); each hemisphere was treated as one sample. (CG1: CPP + training, *n* = 18, Res-relief, *n* = 18; CG2: CPP + training, *n* = 18, Res-relief *n* = 18; LSD: CPP + training, *n* = 15, Res-relief, *n* = 15; MS: CPP + training, *n* = 6, Res-relief *n* = 9; dNacMed: CPP + training *n* = 18, Res-relief, *n* = 18; NacLat: CPP + training, *n* = 18, Res-relief, *n* = 18; CA1: CPP + training, *n* = 18, Res-relief, *n* = 18; DG: CPP + training, *n* = 18, Res-relief, *n* = 18; CeA: CPP + training, *n* = 18, Res-relief, *n* = 18; M2: CPP + training *n* = 18, Res-relief, *n* = 18; S2: CPP + training *n* = 18, Res-relief, *n* = 18). **(C)** Representative c-Fos immunohistochemistry images of the PFC, LSD, MS, dNacMed, NacLat, CA1, DG, CeA, M2, and S2 in female mice after stress relief (bar = 100 μm) (*n* = 3). **(D)** Numbers of c-Fos-positive cells in the above regions. c-Fos-positive cells were quantified bilaterally (three sections per mouse, three mice per group); each hemisphere was treated as one sample. (CG1: CPP + training, *n* = 18, Res-relief, *n* = 16; CG2: CPP + training, *n* = 18, Res-relief, *n* = 16; LSD: CPP + training, *n* = 18, Res-relief, *n* = 16; MS: CPP + training, *n* = 7, Res-relief, *n* = 7; dNacMed: CPP + training, *n* = 18, Res-relief, *n* = 17; NacLat: CPP + training, *n* = 18, Res-relief, *n* = 16; CA1: CPP + training, *n* = 18, Res-relief, *n* = 18; DG: CPP + training, *n* = 18, Res-relief, *n* = 18; CeA: CPP + training, *n* = 16, Res-relief, *n* = 16; M2: CPP + training, *n* = 18, Res-relief, *n* = 15; S2: CPP + training, *n* = 18, Res-relief, *n* = 18). The data are presented as the mean ± SEM and were analyzed by an unpaired two-tailed Student’s t test. When unequal variances were detected by the F-test, Welch’s correction was applied. ^*^p < 0.05, ^**^p < 0.01, ^***^p < 0.001, ^****^p < 0.0001 versus the CPP + training group

To identify the potential brain regions involved in the regulation of different stress relief responses in male and female mice, we quantified the expression of c-Fos in distinct brain areas following the CPP test. In male mice, restraint stress relief significantly reduced the number of c-Fos-positive cells, particularly in regions associated with emotion, reward, fear processing and sensory activity, such as the hippocampus (CA1, t_(34)_ = 3.93, *p* = 0.0004; DG, t_(34)_ = 3.17, *p* = 0.0032), central amygdala (CeA, t_(34)_ = 5.97, *p* < 0.0001), motor cortex (M2, t_(34)_ = 0.6385, *p* = 0.5275), and secondary somatosensory cortex (S2, t_(34)_ = 5.738, *p* < 0.0001). Nonetheless, stress relief did not result in any alterations in c-Fos expression within the prefrontal cortex (CG1, t_(34)_ = 1.88, *p* = 0.069; CG2, t_(34)_ = 1.99, *p* = 0.055), lateral septal nucleus, dorsal part (LSD, t_(28)_ = 0.0642, *p* = 0.949), medial septal nucleus (MS, t_(13)_ = 1.646, *p* = 0.1238), nucleus accumbens lateral shell (NacLat, t_(34)_ = 0.0704, *p* = 0.944) or dorsomedial shell (dNacMed, t_(34)_ = 1.056, *p* = 0.299) (Fig. 4A–B). In female mice, compared with the CPP + Training group, the Res-relief group presented significantly lower c-Fos expression in the prefrontal cortex (CG1, t_(32)_ = 2.550, *p* = 0.016; CG2, t_(32)_ = 5.244, *p* < 0.0001), dorsal nucleus accumbens medial shell (dNacMed, t_(33)_ = 5.890, *p* < 0.0001), lateral nucleus accumbens (NacLat, t_(27.86)_ = 3.49, *p* = 0.0016), and motor cortex (M2, t_(20.1)_ = 5.226, *p* < 0.0001). No significant differences were observed in the LSD, MS, DG, CeA, or S2 regions (LSD, t_(28.12)_ = 1.565, *p* = 0.1288; MS, t_(12)_ = 0.0104, *p* = 0.9919; DG, t_(20.42)_ = 0.5624, *p* = 0.58; CeA, t_(27.21)_ = 1.042, *p* = 0.3068; S2, t_(34)_ = 0.0982, *p* = 0.9223) between these two groups (Fig. 4C–D). Therefore, the different stress relief responses in male and female mice might be derived from different brain circuits.

**Fig. 5:**
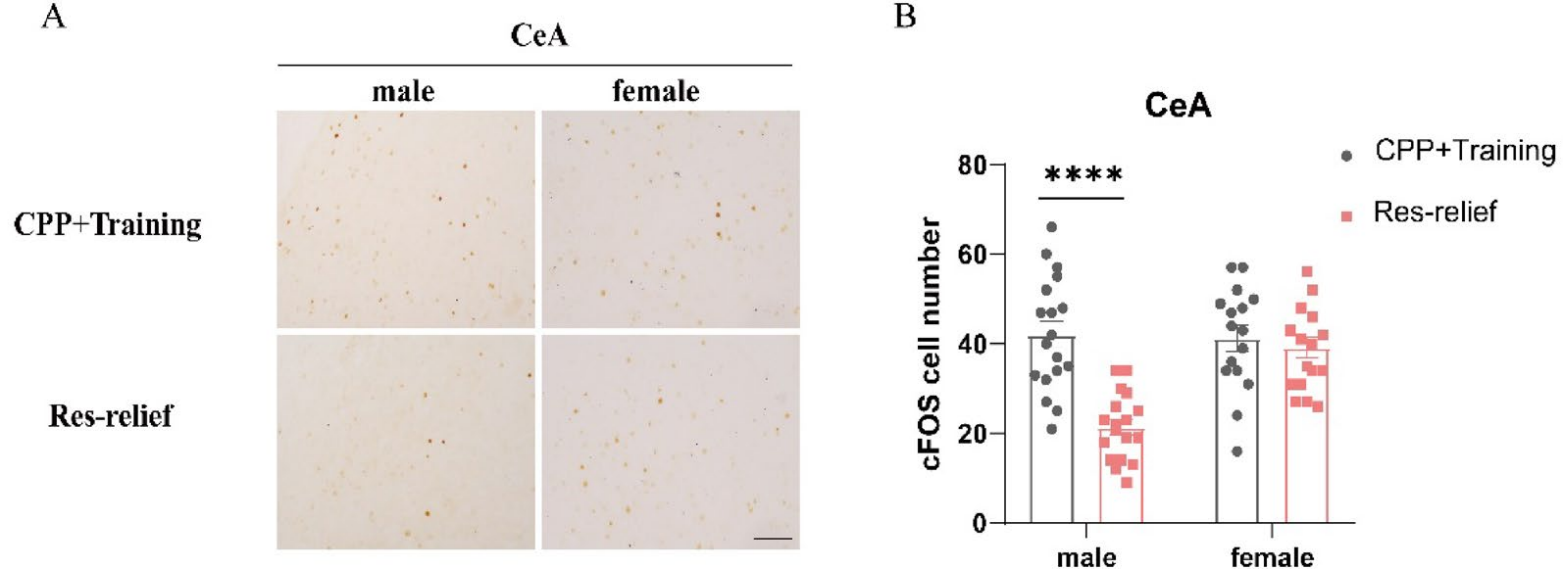
Sex differences in CeA activation following stress relief, with higher c-Fos expression in females than in males. **(A)** Representative c-Fos immunohistochemistry images of the CeA in male and female mice after stress relief (bar = 100 μm). **(B)** Numbers of c-Fos-positive cells in the CeA region in male and female mice. c-Fos-positive cells were quantified bilaterally (three sections per mouse, three mice per group); each hemisphere was treated as one sample. (CPP + training male, *n* = 18; Res-relief male, *n* = 18; CPP + training female, *n* = 16; Res-relief female, *n* = 16). The data are presented as the mean ± SEM and were analyzed by two-way ANOVA followed by Bonferroni’s post hoc correction. ^****^p < 0.001 versus males in the CPP training group

Notably, the different patterns of c-Fos expression in the CeA attracted our attention because a previous study revealed that the CeA is a critical brain region involved in aversive stimulus processing and stress perception [29]. To further clarify the differences in c-Fos expression in the CeA, we extracted the relevant data of c-Fos from Fig. 4 and presented them separately for this region. As shown, we systematically assessed c-Fos expression in the CeA of male and female mice (Fig. 5A). Two-way ANOVA revealed significant main effects of sex (F_(1,65)_ = 11.29, *p* = 0.0013) and restraint stress (F_(1,65)_ = 20.19, *p* < 0.0001), as well as a significant sex × stress interaction (F_(1,65)_ = 13.50, *p* = 0.0005). Bonferroni’s multiple comparisons test revealed that compared with control male mice, stressed male mice exhibited a markedly reduced number of c-Fos-positive cells in the CeA (*p* < 0.0001), whereas stressed and control female mice displayed comparable c-Fos expression levels (Fig. 5B). We speculated that the CeA might be involved in the regulation of sex-specific stress relief responses. However, we also noted that stressed female mice exhibited a markedly reduced number of c-Fos-positive cells in the MeA compared with control females (t = 3.217, *p* = 0.0041), whereas no significant differences in c-Fos expression were observed between stressed and control male mice (t = 1.118, *p* = 0.5358) (Supplementary Figures S1C-D), which is not the case in the BLA (Supplementary Figures S1A-B).

### Chemogenetic activation of the central amygdala abolishes the stress relief response in male mice

**Fig. 6:**
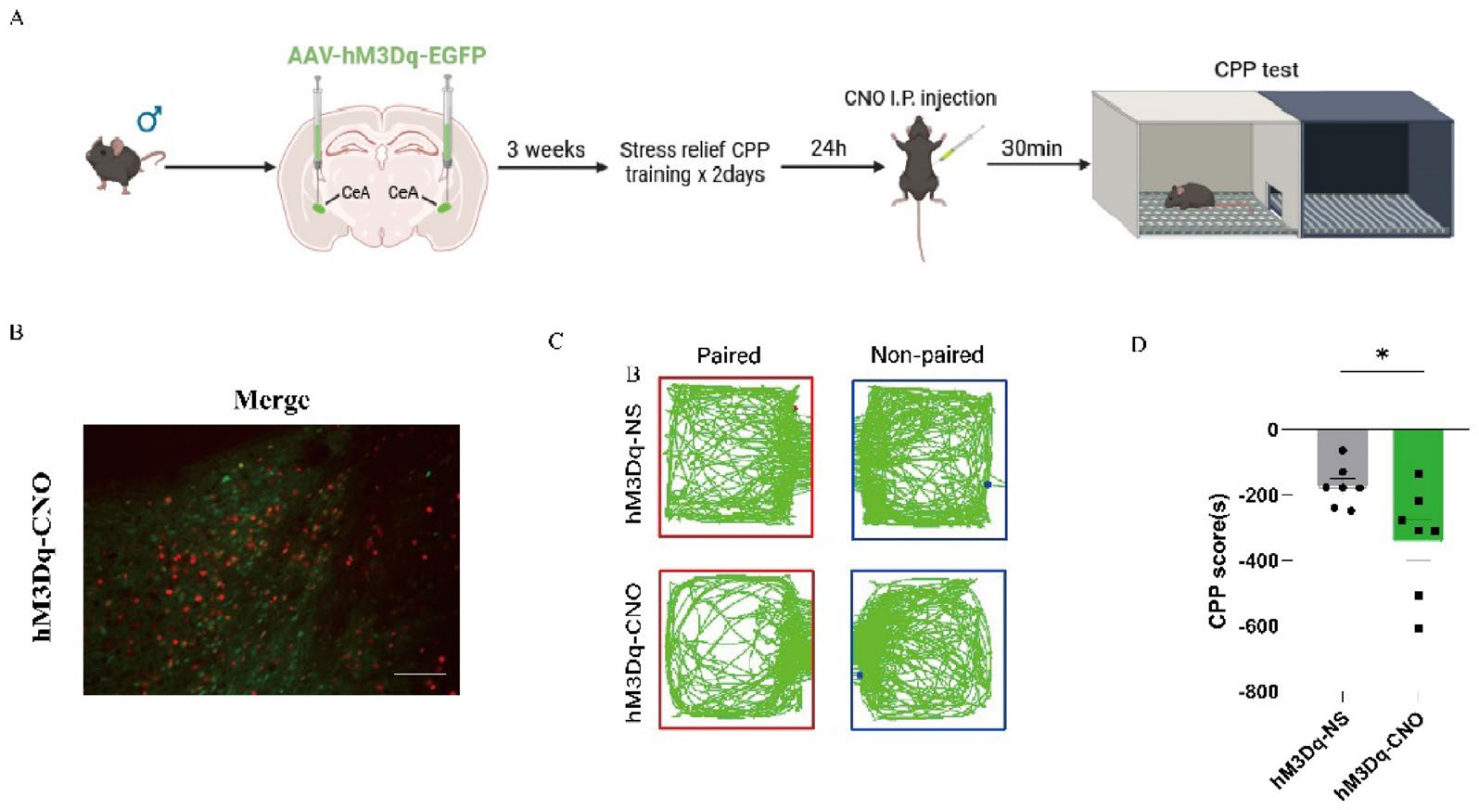
Chemogenetic activation of the central amygdala abolishes stress relief in male mice. **(A)** Schematic timeline of chemogenetic activation in the restraint-relief CPP test. **(B)** Representative images of chemogenetic virus and c-Fos fluorescence colabeling (bar = 100 μm). **(C)** Trajectory map of hM3Dq-NS and hM3Dq-CNO mice in the CPP test. **(D)** CPP scores of hM3Dq-NS and hM3Dq-CNO mice (hM3Dq-NS = 7, hM3Dq-CNO = 7). The data are presented as the mean ± SEM and were analyzed by an unpaired two-tailed Student’s t test. When unequal variances were detected by the F-test, Welch’s correction was applied. ^*^p < 0.05 versus hM3Dq-NS

To explore the potential role of the CeA in the regulation of the stress relief response in mice, we injected a chemogenetic virus (AAV2/9-hSyn-hM3Dq) into the central amygdala of male mice to manipulate neuronal activation in this region (Fig. 6A). The fluorescence colabeling of c-Fos and EGFP confirmed the successful expression of the chemogenetic virus (Fig. 6B). Notably, we observed that compared with vehicle-treated male mice, CNO-treated male mice exhibited significantly lower CPP scores (t_(7.74)_ = 2.476, *p* = 0.0393) (Fig. 6C–D).

### Chemogenetic Inhibition of the central amygdala facilitates stress relief behavior in female mice

**Fig. 7:**
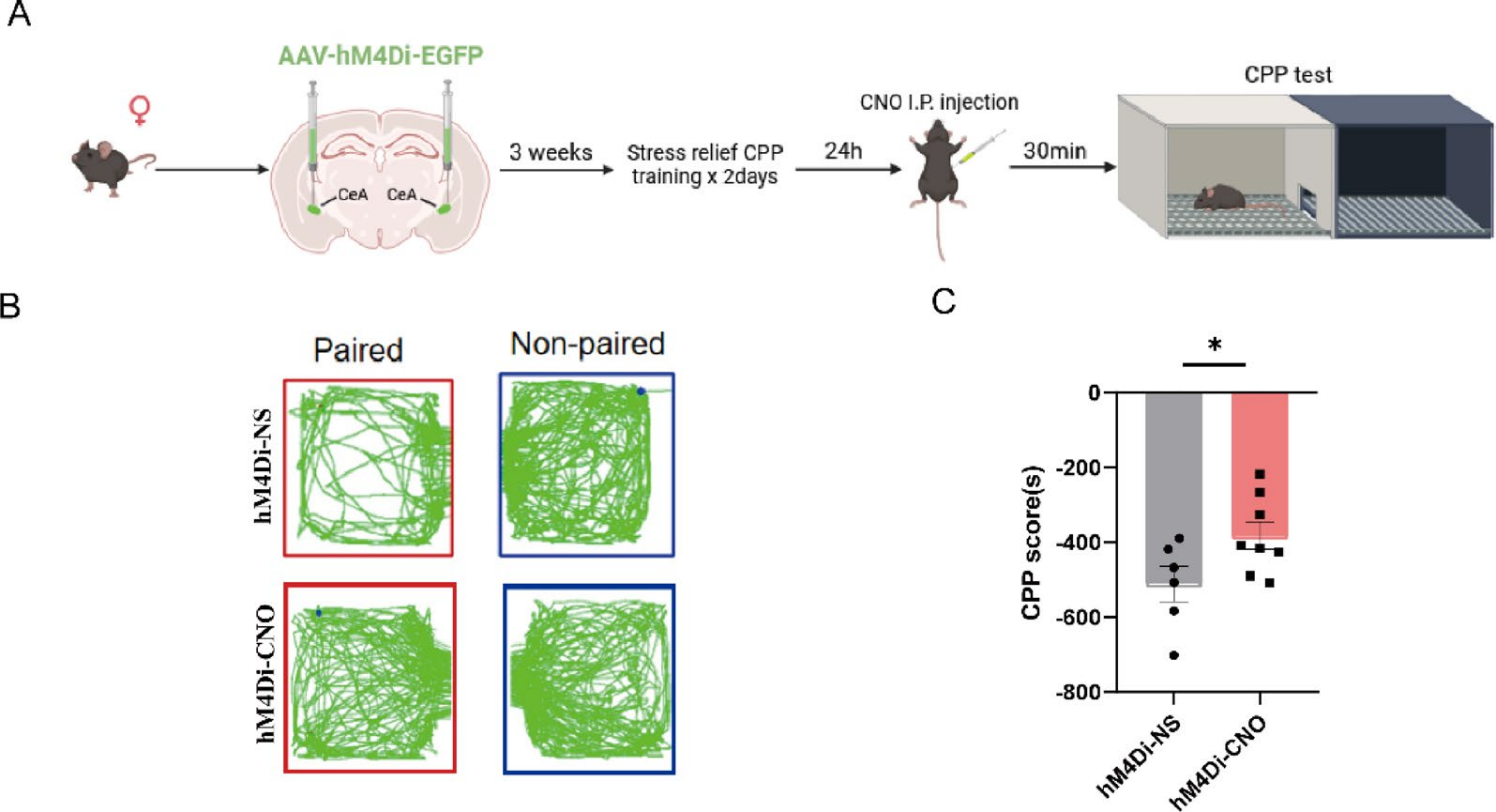
Chemogenetic inhibition of the central amygdala reverses stress relief behavior in female mice. **(A)** Schematic timeline of chemogenetic inhibition in the restraint-relief CPP test. **(B)** Trajectory map of hM4Di-NS and hM4Di-CNO mice in the CPP test. **(C)** CPP scores of hM4Di-NS and hM4Di-CNO mice (hM4Di-NS = 6; hM4Di-CNO = 8). The data are presented as the mean ± SEM and were analyzed by an unpaired two-tailed Student’s t test. ^*^p < 0.05 versus hM4Di-NS

Because chemogenetic activation of the CeA in male mice suppressed the stress relief response, we next sought to determine whether chemogenetic inactivation of the CeA would facilitate this behavior in female mice using a chemogenetic inhibition virus (AAV2/9-hSyn-hM4Di) (Fig. 7A). We found that compared with vehicle-treated female mice, CNO-treated female mice that were reinstated had significantly greater CPP scores (t_(12)_ = 2.196, *p* = 0.0485) (Fig. 7B–C). And the potential off-target effect of Chemogenetic manipulation of CeA neurons was also validated (Supplementary Figures S2A-E**)**.

## Discussion

Understanding the mechanisms that regulate the stress relief response may provide important insights into the function of the natural reward system in the brain and offer novel strategies to combat disorders and diseases characterized by a dysregulated brain reward system. Our findings reveal a sex-specific effect in the stress relief response, where females but not males are less likely to experience a stress relief response, which is a previously unknown sex-dependent behavior. Using a screening approach based on the detection of c-Fos, we found that CeA neurons are inactivated during stress relief in male mice but are highly activated in female mice. Moreover, chemogenetic activation of these neurons is sufficient to reduce stress relief responses in male mice, whereas chemogenetic inactivation of these neurons can facilitate stress relief responses in female mice. Thus, the differences in neuronal activation in the CeA may provide a concrete anatomical substrate underlying the sexual dimorphism in stress relief responses in mice. These data highlight an unappreciated sex-specific effect in the stress relief response and reveal a causal role of amygdala neurons in the regulation of this behavior in rodents.

### Sex differences in the stress relief response

With increasing interest in exploring sex differences in rodent behaviors, an increasing number of laboratories have reported sex differences that were not reported in previous studies [30–32]. Stress in daily life often leads to negative emotions, whereas the relief following the cessation of stress can produce a sense of release and pleasure, suggesting that opposing emotions can generate new motivational behaviors, which might be due to the natural reward system of the brain [33]. Using a stress relief experimental paradigm [18], we measured the stress relief responses of both male and female mice. In agreement with the findings of a previous study [18], we indeed found that male mice showed stress relief responses in both the restraint stress paradigm and the foot-shock stress paradigm. However, we found that female mice exhibited significantly fewer stress relief responses in these paradigms, as indicated by the same CPP scores as those of the naive female mice. Considering the sex differences in the regulation of the hypothalamic–pituitary–adrenal axis (HPA) and its hormonal reactivity to stressors [34, 35], we speculate that the different stress relief responses observed in the present study might be due to differences in stress sensitivity. To exclude the potential effects of different stress sensitivities on these behaviors, we also used different intensities of stress stimuli with different foot shock intensities. To our surprise, the female mice still demonstrated the same CPP score, as both low and high levels of electric foot shock did not induce place preference in the light box. Given that we did not find any difference in female mice that experienced mild or strong stress stimuli, we consider that other factors might influence this behavior. However, determining the CORT level before and after the CPP test in male and female mice would be more rigorous.

### Sex differences in the stress relief response are not dependent on estrous cycle phase

Both estradiol and testosterone are powerful steroid hormones that impact brain function in numerous ways [36, 37]. Circulating sex hormones can influence neurotransmitter systems relevant to reward [36–38]. Thus, one potential explanation for the behavioral differences between female and male mice is the effect of the estrous cycle. In fact, the stress relief conferred by “comfort foods” varies across the menstrual cycle in women [39, 40], suggesting the potential for important sex- and cycle-dependent stress relief responses. To our surprise, we observed that the low levels of stress relief responses in female mice were not due to the P/E estrous cycle stage, as indicated by the same CPP scores at different estrous cycle stages. We acknowledge that the limited representation across estrous phases and stress conditions (P/E: *n* = 2–4) might have led to some bias in the present data; however, we found that females had comparable CPP scores on D1/D2 in both the restraint stress and foot-shock stress paradigms, which was not the case in the P/E. Notably, similar to those of ovarian hormones, testosterone levels are not stable, as testosterone levels vary between group-housed male mice because of social hierarchy [41] and fluctuate on both daily (humans and rodents) and monthly cycles [42–44], which was not monitored in this study. Taken together, our data suggest that the sex differences in stress relief behavior are not dependent on specific hormonal phases, although the potential modulatory roles of circulating sex hormones cannot be completely excluded.

### The CeA is involved in sex regulation of the stress relief response

Sex differences have been observed in different brain regions [45–48] as well as in the prevalence of psychological disorders [49, 50], although the reasons for these sex biases remain unknown. In fact, limbic structures, including the amygdala and hippocampus, are critical for coordinating behavioral responses to stressors [51, 52], all of which show sex-dependent patterns of reactivity to stress [53, 54]. Exposure to aversive, stressful stimuli leads to a multisystem biological response that prepares an organism for potential harm through physiological, behavioral, hormonal, and autonomic responses [55, 56]. These stress-relieving effects may be partially explained by their ability to alter the long-term activity of neurocircuitry in reward- and stress-regulatory brain regions, such as the NAc and amygdala [18, 57]. Immediate-early genes (IEGs), such as c-Fos, are genes that are rapidly induced in response to neuronal stimulation, and IEG proteins play important roles in neural plasticity and memory [58, 59]; thus, brain-wide IEG imaging in rodents can detect coordinated activation with high spatial resolution [60]. To identify the brain regions involved in the stress relief response, we quantified neuronal activation, which is a marker of neuronal activity, in several brain regions using c-Fos immunohistochemistry [61, 62]. We measured changes in c-Fos expression in 8 regions of interest, which are composed of 11 different brain subregions, most of which are limbic structures. Initially, we focused on the NAc because this region plays critical roles in modulating the stress relief response in rodents [18]; however, we observed no change in the NAc in male mice, which is inconsistent with the findings of a previous study [18]. This discrepancy may stem from temporal windows between calcium imaging techniques and c-Fos detection approaches, as c-Fos mapping provides an excellent description of spatial patterns of activation but cannot adequately detect rapid differences in temporal patterns of coordinated activity [63]. However, we indeed found a reduced number of c-Fos-positive cells in the NAc of female mice, indicating differences in the motivation and reward system, which is consistent with the findings of a previous study [64]. Notably, we observed that the amygdala, especially the CeA, contained low levels of c-Fos in a stress relief assay in male mice, whereas this parameter was unchanged in female mice. Sex differences have been observed within the amygdala in terms of size and the proportion of subnuclei across ages, which are functionally distinct [47]. Interestingly, when looking at the response of the amygdala to familiar negative images in humans, it has been observed that adult females show a sustained amygdala response, whereas males do not [65]. With respect to the present data, we indeed found that the number of c-Fos-positive cells was much more stable in the CeA of female mice than in that of male mice, as indicated by the same level of c-Fos under basic conditions and in a subsequent stress relief assay. Although psychological stressors reliably activate amygdala neurons, rewarding stimuli also activate amygdala neurons. We believe that the inactivation of the amygdala might be due to the safe signal provided by the cessation of stress, which differs from the findings of previous studies [66–68]. To corroborate the involvement of CeA neurons in the stress relief response, we chemogenetically activated CeA neurons and found that the activation of CeA neurons effectively abolished the stress relief response in male mice. Importantly, we observed that chemogenetic inactivation of CeA neurons in female mice facilitated the stress relief response, as indicated by increased CPP scores. Although a role for the CeA in modulating responses to reward-predictive cues and safety signals has been reported [69, 70], our data revealed for the first time that CeA neurons are involved in the stress relief response, which also has sex differences. The observed sex differences in CeA neuronal activity during stress relief could be attributed to several factors, such as structural and functional differences in the CeA and its connectivity with other brain regions involved in stress and fear, which might underlie these sex-specific patterns of activity [71]. We also realized that the role of other sub-regions of amygdala in our experiment should not be excluded. The basolateral nucleus (BLA) encodes emotional events with reference to their particular sensory-specific features, whereas the central nucleus (CeA) encodes their more general motivational or affective significance [72]. Plasticity in the BLA supports the formation and storage of an association between environmental information and shock and passes this information to the CeA, whose efferents to the ventral periaqueductal gray trigger the expression of fear as indexed by conditional freezing [73–76]. Importantly, we observed no alteration of c-fos in the basolateral amygdala (BLA), where c-Fos expression remained comparable across stress conditions and sexes. In contrast, the medial amygdala (MeA) displayed a distinct pattern, with stressed female mice showing a significant reduction in c-Fos expression compared with control females, while no significant change was observed in males. Given the established role of the MeA at the intersection of social cue recognition and connects with limbic structures that control a broad repertoire of social behaviours and physiology, including social hierarchical interactions [77, 78], stress [79, 80], which notably exhibits significant sex differences in both structure and function, contributing to differences in social behavior and emotional responses between males and females [81]. This finding suggests that MeA may also contribute to sex-specific modulation of sex differences in stress relief behavior, which needs further investigation. In addition, we also examined hippocampal and sensory cortical regions, which are closely associated with emotions and stress regulation [82]. In males, stress relief was accompanied by a significant reduction in neuronal activity within these regions. In contrast, females exhibited increased activation following stress relief, suggesting sex-specific recruitment of hippocampal and cortical circuits in coping with stress. These results indicated that these brain regions might also be involved in this process, which requires further investigation. These neural differences could reflect distinct strategies for processing and adapting to stress between males and females, as supported by research showing sex-dependent variability in stress-induced neural activity and subsequent behavioral outcomes [83]. Taken together, our whole-brain c-Fos mapping and functional manipulations supported that the CeA was required for this stress relief response. It should be noted that the hSyn promoter used in this study could not provide cell type specificity and therefore drives broad neuronal expression within the CeA. Although this approach allowed us to reliably modulate regional CeA activity and assess its overall contribution to the stress relief response, future studies employing cell type–specific strategies (e.g., Cre-dependent AAVs in CRH-Cre or GAD-Cre mice) will be necessary to dissect the precise roles of distinct CeA neuronal subpopulations. Although no behavioral alterations were observed in control animals in the present study, we also acknowledge that the use of CNO as a DREADD ligand may produce nonspecific effects, especially when saline is used as the only control.

### Perspectives and significance

Overall, these findings clearly revealed sex differences in the stress relief response in mice, with male mice being more sensitive. Moreover, chemogenetic manipulations supported that the CeA was required for this stress relief response. Although our model cannot completely recapitulate human stress relief or make direct comparisons, we can draw parallels from similarities in behavioral domains. Answering these questions will provide possible targets for differential therapeutic intervention in men and women.

Although these data are exciting and informative, some limitations should be acknowledged. We used c-Fos expression to explore the involvement of other brain regions in response to stress. Given its expression kinetics, the efficiency of c-Fos in accounting for previous stress exposure and the different phases of our protocol is limited. Notably, estrous cycle assessments by vaginal lavage were restricted to the days of sample collection, as opposed to those occurring daily throughout the entire experiment. We chose to limit the number of vaginal lavages to avoid the possibility that the stress associated with repeated daily lavages could confound the behavioral and hypothalamic–pituitary–adrenal (HPA) axis responses to stress. In addition, while we aimed to restrict viral expression to the CeA, occasional leakage to adjacent regions may have occurred. This potential spread should be considered when interpreting the region-specific effects observed in our study. Although chemogenetic activation of the CeA effectively abolished stress relief behavior, the specific cell types and projection pathways mediating this effect were not identified.

## Conclusions

In summary, our data have significant implications for our understanding of the stress relief response, especially in the context of sex differences, and reveal a causal role of CeA neurons in the regulation of this behavior in male mice. To ensure that the translation of preclinical research applies to both male and female individuals, the current study emphasizes the vital consideration of sex differences and the inclusion of mixed-sex cohorts in studies utilizing the stress relief model. Given the growing interest in understanding the molecular and cellular mechanisms underlying brain function and behavior, particularly in the context of sex differences, our findings provide valuable insights into the field of neuroscience.

## Supplementary Information

Supplementary Material 1

## Data Availability

No datasets were generated or analysed during the current study.
