# Supplementary material for: Involvement of the central amygdaloid nucleus in the regulation of sex differences in the stress relief response in mice
Source: Biol Sex Differ. 2026 Jan 16;17:27. doi: 10.1186/s13293-025-00819-z (PMC12892754; doi:10.1186/s13293-025-00819-z)
Supplement: Supplementary file 1 — Supplementary Material 1 [file 13293_2025_819_MOESM1_ESM.doc]

**1. Supplementary Figures S1 Sex-dependent c-Fos expression patterns in the basolateral (BLA) and medial (MeA) amygdala following stress relief in male and female mice**

To further characterize sub-region of amygdala activation, we also quantified c-Fos expression in the BLA and MeA following stress relief in both male and female mice. In BLA, Two-way ANOVA revealed significant main effects of sex (F (1, 59) = 9.940, p=0.0025) but not restraint stress (F (1, 59) = 1.317, p=0.2557). Bonferroni’ s multiple comparisons test revealed that both male and female, stressed or control mice, exhibited comparable levels of c-Fos expression in these regions (male: t=0.5237, p>0.9999; female: t=1.072, p=0.5759) (Fig.S1A-B). However, in MeA, Two-way ANOVA revealed significant main effects of sex (F (1, 63) = 20.42, p<0.0001) and restraint stress (F (1, 63) = 9.778, p=0.0027). Bonferroni’ s multiple comparisons test showed that stressed female mice exhibited a markedly reduced number of c-Fos-positive cells in the MeA compared with control females (t=3.217, p=0.0041), whereas no significant differences in c-Fos expression were observed between stressed and control male mice (t=1.118, p=0.5358) (Fig.S1C-D).


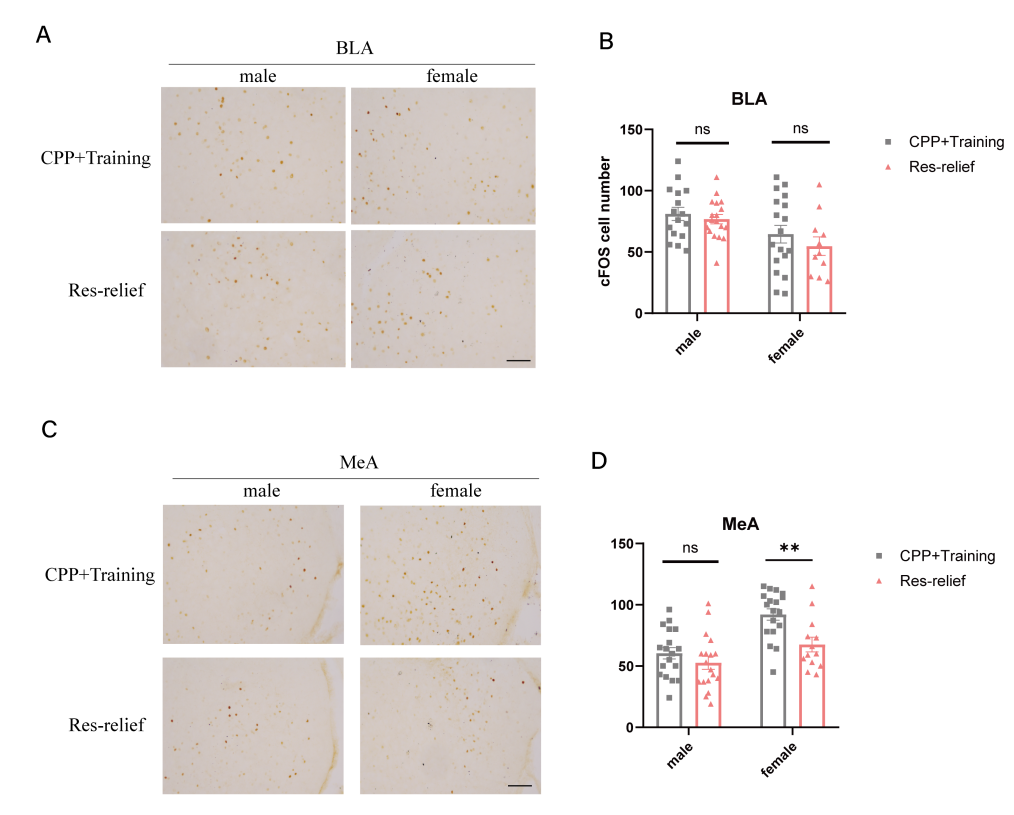


**Fig. S1**. Sex-dependent c-Fos expression patterns in the basolateral (BLA) and medial (MeA) amygdala following stress relief.(A) Representative c-Fos immunohistochemistry images of BLA in male and female mice after stress relief (bar = 100 µm). (B) The number of c-Fos positive cells of male and female mice in BLA region. c-Fos positive cells were quantified bilaterally (three sections per mouse, three mice per group); each hemisphere was treated as one sample. (CPP+training-male n=16, Res-relief-male n=18; CPP+training-female n=18, Res-relief-female n=11). (C) Representative c-Fos immunohistochemistry images of MeA in male and female mice after stress relief (bar = 100 µm). (D) The number of c-Fos positive cells of male and female mice in MeA region. c-Fos positive cells were quantified bilaterally (three sections per mouse, three mice per group); each hemisphere was treated as one sample. (CPP+training-male n=18, Res-relief-male n=18; CPP+training-female n=18, Res-relief-female n=13). Data are mean ± SEM, analyzed by Two-way ANOVAs followed by Bonferroni’s post hoc test. ^**^p < 0.01 versus CPP training-female.

**2. Supplementary Figures S2 Validation of the potential off-target effect of Chemogenetic manipulation of CeA neurons**

To assess potential off-target effect of chemogenetic manipulation of CeA neurons, we examined c-Fos expression in adjacent amygdala nuclei following CNO challenge (Fig.S2A-B). Neither hM3Dq nor hM4Di altered c-Fos expression in the BLA (Fig.S2C-F), whereas partial MeA activation was observed in a small subset of hM3Dq-treated animals, which might be due to viral leakage beyond the CeA boundaries (Fig.S2D).


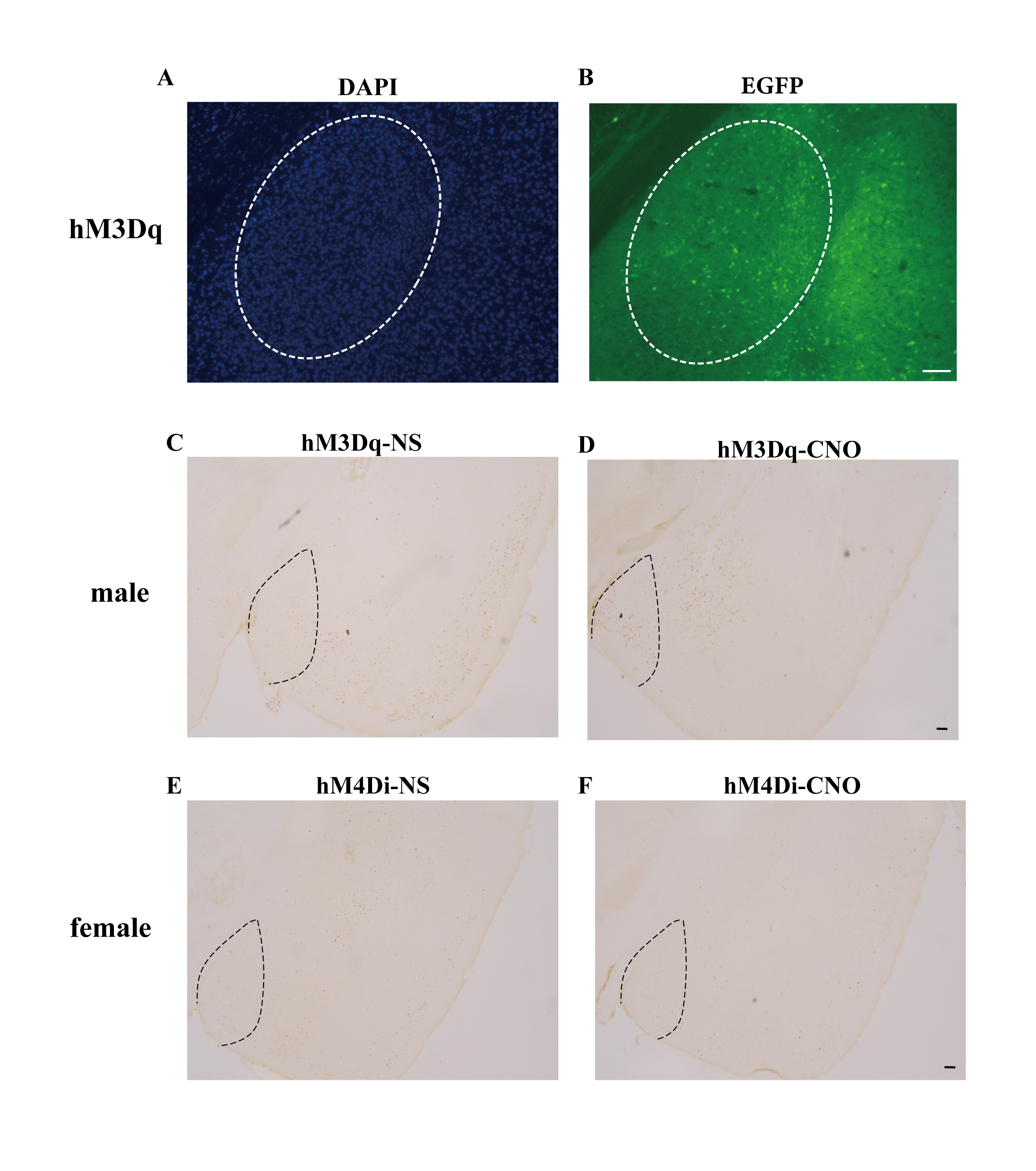


Fig.S2. Representative viral expression and c-Fos expression following Chemogenetic manipulation of CeA neurons. (A) DAPI staining following Chemogenetic injection (bar=200μm). (B) EGFP fluorescence indicating chemogenetic activation (bar=200μm). (C) Representative c-Fos immunohistochemistry images of BLA, MeA in hM3Dq-NS male mice (bar=500μm). (D) Representative c-Fos immunohistochemistry images of BLA, MeA in hM3Dq-CNO male mice (bar=500μm). (E) Representative c-Fos immunohistochemistry images of BLA, MeA in hM4Di-NS female mice (bar=500μm). (F) Representative c-Fos immunohistochemistry images of BLA, MeA in hM4di-CNO female mice (bar=500μm).
